# Impact of Biological Therapies on the Immune Response after Pneumococcal Vaccination in Patients with Autoimmune Inflammatory Diseases

**DOI:** 10.3390/vaccines9030203

**Published:** 2021-02-28

**Authors:** Patricia Richi, Jose Yuste, Teresa Navío, Laura González-Hombrado, Marina Salido, Israel Thuissard-Vasallo, Ana Jiménez-Díaz, Jesús Llorente, Laura Cebrián, Leticia Lojo, Martina Steiner, Tatiana Cobo, María Dolores Martín, Marta García-Castro, Patricia Castro, Santiago Muñoz-Fernández

**Affiliations:** 1 Rheumatology Unit, Hospital Universitario Infanta Sofía San Sebastián de los Reyes, 28702 Madrid, Spain; ajimenezd@salud.madrid.org (A.J.-D.); martina.steiner@salud.madrid.org (M.S.); mtatiana.cobo@salud.madrid.org (T.C.); santiago.munoz@salud.madrid.org (S.M.-F.); 2 School of Medicine, Universidad Europea, 28670 Madrid, Spain; 3 Spanish Pneumococcal Reference Laboratory, National Center for Microbiology, Instituto de Salud Carlos III, 28220 Madrid, Spain; 4 Centro de Investigación Biomédica en Red de Enfermedades Respiratorias (CIBERES), Instituto de Salud Carlos III, 28029 Madrid, Spain; 5 Rheumatology Unit, Hospital Universitario Infanta Leonor, 28031 Madrid, Spain; mteresa.navio@salud.madrid.org (T.N.); laura.cebrian@salud.madrid.org (L.C.); leticia.lojo@salud.madrid.org (L.L.); 6 Rheumatology Unit, Hospital del Tajo, Aranjuez, 28300 Madrid, Spain; lgonzalez.hdoc@salud.madrid.org (L.G.-H.); gamarta@hotmail.com (M.G.-C.); 7 Rheumatology Unit, Hospital Universitario Infanta Cristina, Parla, 28981 Madrid, Spain; marina.salido@salud.madrid.org (M.S.); patricia.castro@salud.madrid.org (P.C.); 8 School of Doctoral Studies & Research, Universidad Europea, 28670 Madrid, Spain; israeljohn.thuissard@universidadeuropea.es; 9 Pharmacy Department, Hospital Universitario Infanta Sofía, San Sebastián de los Reyes, 28702 Madrid, Spain; jesus.llorente@salud.madrid.org; 10 BR Salud Laboratories, Bacteriology Department, San Sebastián de los Reyes, 28702 Madrid, Spain; dmrodrigo@salud.madrid.org

**Keywords:** PPV23, PCV13, OPKA titers, invasive pneumococcal disease, biological therapy, anti-TNFα, rituximab, tocilizumab, autoimmune inflammatory diseases

## Abstract

Patients with different autoimmune inflammatory diseases (AIID) on biological therapy are at risk of pneumococcal disease. Adults with inflammatory arthropathies, connective tissue diseases, psoriasis, or inflammatory bowel disease on biological therapy such as anti-TNFα, rituximab, tocilizumab, abatacept, or anakinra were included in this study. Patients completed a protocol combining the pneumococcal vaccines PCV13 and PPV23. Immune response against pneumococcal serotypes 1, 3, 7F, 14, 19A, and 19F were assessed evaluating functional antibodies by an opsonophagocytosis killing assay (OPKA). In this study, 182 patients with AIID completed the sequential vaccination protocol. Patients on etanercept tended to achieve OPKA titers against a larger number of serotypes than the rest of patients on other biological therapies, while adalimumab was associated to a lower number of serotypes with OPKA titers. Rituximab was not associated with a worse response when compared with the rest of biological agents. Not glucocorticoids, nor synthetic disease-modifying antirheumatic drugs, interfered with the immune response. OPKA titers against serotype 3 which is one of the most prevalent, was obtained in 44% of patients, increasing up to 58% in those on etanercept. Hence, almost 50% of patients on biological therapy achieved functional antibodies after the administration of a complete pneumococcal vaccination protocol.

## 1. Introduction

People with autoimmune inflammatory diseases (AIID) have an increased risk of invasive pneumococcal disease (IPD) and nonbacteremic pneumonia. The relative risk (RR) has been described between 2.5 and 4.9 in rheumatoid arthritis patients (RA) and between 5 and 14.2 in patients with systemic lupus erythematosus (SLE), being much higher in adults under 50 years old [1,2,3]. Pneumococcal vaccination reduces the severity of these infections and is associated to lower hospital admission rates, less visits to emergency units and reduced IPD rates in patients with autoimmune inflammatory rheumatic diseases (AIIRD) treated with anti TNF-α drugs [4]. Pneumococcal vaccination in adults with AIIRD was included in the first European League against Rheumatism (EULAR) recommendations in 2011 [5], keeping this vaccine strategy in 2019 for patients with AIIRD receiving immunosuppressive treatment [6]. EULAR recommendations agree with the Advisory Committee on Immunization Practices (ACIP) guidelines, that encourage routine use of 13-valent pneumococcal conjugate vaccine (PCV13) for adults aged ≥ 19 years with immunocompromising conditions (including those with diseases requiring treatment with immunosuppressive drugs), in addition to the 23-valent pneumococcal polysaccharide vaccine (PPV23) [7]. In Spain, the country where the present study was carried out, 18 different Scientific Societies, including Spanish Society of Rheumatology, elaborated a consensus document on pneumococcal vaccination in adults at risk by age and underlying clinical conditions. This document includes AIIRD as ones of those clinical conditions candidates for pneumococcal vaccination coverage [8].

Although AIID is one of the clinical conditions in which pneumococcal vaccination is recommended by international Scientific Societies and guidelines, the vaccination coverage remains low, and it varies from 6% to 62% [9,10]. The reasons for not being vaccinated include fear of adverse effects, absence of patient information by the primary-care doctors and rheumatologists and physicians’ concerns because treatment with a biological agent has been associated to an impaired immune response, that may affect the efficacy of the vaccines in these patients [11]. On the other hand, data on efficacy and/or effectiveness of pneumococcal vaccination in patients with AIID is difficult to interpret, taking into account that the scarce number of studies that analyze this subject differ in the vaccines used and the variables studied.

The aim of our study was to investigate different aspects of the immunological response to pneumococcal vaccination in patients with AIID that are receiving biological treatment. One goal was to evaluate the influence on the immune response by the biological therapy in order to demonstrate if the effect depends on the type of biological DMARD (disease modifying antirheumatic drug) used in routine clinical practice. We also wanted to know if concomitant treatment with glucocorticoids and/or synthetic DMARDs, as well as demographic data, diagnosis and a history of previous biological treatment, affect the vaccine response.

## 2. Materials and Methods

### 2.1. Participants

**Figure 1:**
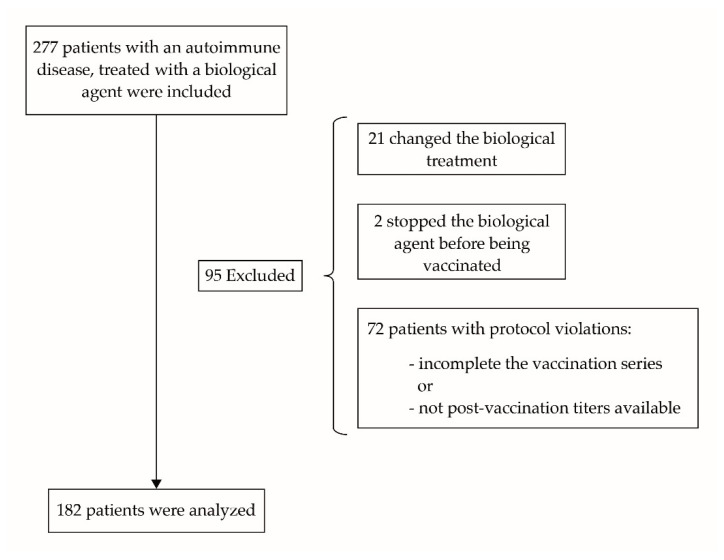
Flow diagram of patients included, excluded and finally analyzed.

A noninterventional, multicenter, cohort study was designed to evaluate the immune response to *Streptococcus pneumoniae* vaccination in patients with AIID attending the Rheumatology, Dermatology or Gastroenterology services of the four University hospitals (Infanta Sofia, Madrid, Spain; Infanta Leonor Madrid, Spain; El Tajo, Madrid, Spain and Infanta Cristina, Madrid, Spain) that participated in the study. Patients that fulfilled the inclusion criteria were invited to enroll in the study. The recruitment period started in October 2014 and the follow-up period finished when the last serological test was performed, at least 4 weeks after the last vaccine was administrated. In total 277 subjects were included at the beginning of the study, 182 of which were analyzed. A flow diagram of selected patients is shown in Figure 1.

Patients older than 18 years, suffering from an AIIRD such as rheumatoid arthritis (RA), spondyloarthritis (SpA), psoriatic arthritis (PsA), undifferentiated arthritis (UA) or connective tissue diseases (CTD), psoriasis (Pso), or inflammatory bowel disease (IBD) were included. In addition, patients had to be on current biological treatment, with an antitumor necrosis factor (anti-TNFα) or other biological agent like, rituximab, tocilizumab, abatacept, or anakinra. Diagnosis and treatments accepted are explained in the results section. The rheumatologists in charge, depending on the patients’ characteristics and clinical parameters, prescribed the biological agent administered to every patient. Patients who stopped or changed their biological DMARD between the basal and the final serological test were excluded from the analysis. Concomitant treatment with synthetic DMARDs and/or glucocorticoids was admitted. Information about if participants had received previous biological agents different from that one they were receiving upon inclusion was recorded. Exclusion criteria included pregnancy, history of known allergy to any of the vaccine components, or an active infection. All participants provided written informed consent. The study did not include Patient and Public Involvement.

### 2.2. Ethical Approval Code

The Clinical Research Ethics Committee of University Hospital La Paz in Madrid, Spain, approved the study (authorization codes HUIGRI-2014-01, HULP: PI-1832).

### 2.3. Vaccination, Immune Response and Microbiology Data

**Figure 2:**
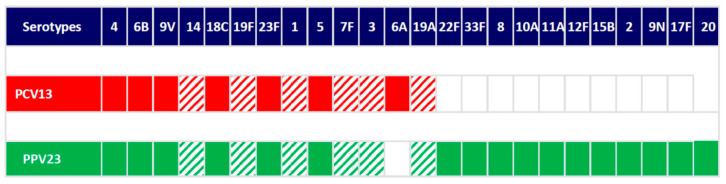
Serotypes included in PCV13 (red color) and PPV23 (green color) and serotypes evaluated for opsonophagocytosis killing assays (hatched). Nonvaccine serotypes (white color).

Vaccination status was recorded from immunization databases of the Primary Care Centres. Patients completed a protocol combining PCV13 and PPV23 following international recommendations [5,6,7]. Blood samples were collected when entering the study and at least 4 weeks after the last vaccine was given. The immune response was evaluated using an opsonophagocytosis killing assay (OPKA) using human HL-60 cells differentiated to neutrophils [12]. The assay required the counting of viable colony forming units (CFU), heat-inactivation of patient’s sera at 56 °C for 30 min in order to avoid variations in phagocytosis mediated by different complement levels among patient’s sera and the use of baby rabbit complement as a source of complement components. Opsonization was performed in 96-microwell plates by incubating 10 µL of bacteria with 20 µL of human serum during 30 min at 37 °C with shaking followed by incubation for 45 min using 20 µL of baby rabbit complement and 40 µL of HL-60 in a 400:1 proportion cells:bacteria. Finally, different dilutions were cultured on blood agar plates to determine the number of viable bacteria after the opsonophagocytosis process. Opsonophagocytic titer (OT) was defined as the serum dilution that kills 50% of bacteria regardless the biological drug and the concomitant treatment used. Values of ≥8 fold increase rise in the OT were considered as a positive response [13,14]. We assessed the immune response to serotypes 1, 3, 7F, 14, 19A, and 19F. The criteria for selecting these serotypes were based in their high epidemiological incidence rates and invasiveness. Serotypes included in both vaccines, PCV13 and PPV23, and the six serotypes selected for OPKA are shown in Figure 2.

### 2.4. Statistics

For the descriptive analysis, the absolute (n) and relative (%) frequencies were used to express the qualitative variables. Mean ± standard deviation (SD) or median (interquartile range; IQR) were used to express the quantitative variables according to the normal distribution. The total number of cases per study variable was used to perform the calculations. To test the statistically significant differences of the vaccine response in the different scenarios, either the Chi-square test or Fisher’s exact test were performed for qualitative variables, while either the Student’s T test or Mann–Whitney U test was used for quantitative variables, according to the normality test. A linear regression analysis was used to evaluate the effects of sex, age and previous biological treatment on the immune response. Statistical significance was considered when the *p*-value was less than the alpha error (5%). Data analysis was performed with IBM SPSS statistics version 21.0 (IBM Corp; Armonk, NY, USA).

## 3. Results

The study analyzed 182 patients. Demographic characteristics (mean age and gender distribution), diagnosis and mean disease duration depending on the diagnosis, as well as biological therapy and concomitant treatments, are shown in Table 1.

Among diagnosis, SpA and RA accounted for 70.4% of the pathologies. Most patients (85.1%) were receiving TNF-α inhibitors. In this group, etanercept and adalimumab were the most frequent treatments, followed by infliximab (Table 1). Synthetic DMARDs were used in 42.3% of the patients, with methotrexate and leflunomide as the preferential drugs (Table 1). Prednisone at different doses was used in 15.4% of patients, with a median (IQR) prednisone dose of 5.0 (2.5) mg per day (Table 1). Pneumococcal vaccination status was registered before admission in the study protocol. Before entering the study, PPV23 had been administered in 115 subjects (63.2%), PCV13 in 21 subjects (12.1%) and only 9 patients (4.9%) had been immunized with both vaccines.

Analysis of the antibody response confirmed that at least one third of the patients achieved OT against each pneumococcal serotype (Table 2).

Serotype 3, which was the most prevalent serotype in adults when patients of this study were recruited [15], showed the largest number of patients with positive OT. The presence of OT against serotype 3 was statistically associated with the detection of OT against serotype 1 (*p* = 0.031), 7F (*p* = 0.004), 19A (*p* = 0.010), and 19F (*p* = 0.0001). Hence, 49% of the patients achieved OT against three or more serotypes resulting in a positive immunological response against ≥ 50% of the serotypes investigated (Table 3).

We studied the influence of age and gender in the number of serotypes with OT after vaccination. We found no correlation between age and the immune response (*p* = 0.907). We also observed no influence of the gender (number of serotypes with OT response in men median (IQR): 2 (2.5) vs. 3 (3) in women, *p* = 0.374). Hence, we did not see differences in the number of serotypes with OT response between the group of patients who had received another biological agent, and those who had been treated with the same biological DMARD since the beginning (median [IQR]: 3 (3) vs. 2 (2), *p* = 0.206). As a result, the regression analysis confirmed that age, gender and having received a previous biological DMARD, did not affect the immune response.

**Figure 3:**
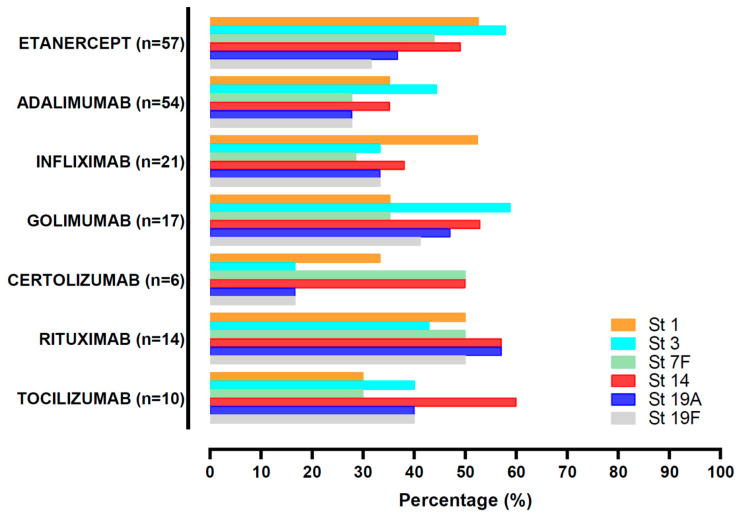
Patients with OT > 50% against each serotype, depending on the biological agent received. Abatacept and anakinra were not included due to the scarce number of patients. St (serotype), OT (opsonization titers).

Among biological DMARDs, etanercept showed a tendency to higher OT response compared to the other therapies (median [IQR]: 3 (2.5), *p* = 0.066) whereas adalimumab had lower OT levels (median (IQR): 1 (2), *p* = 0.015). Rituximab did not show a worse OT response when compared with the other biological agents (median (IQR): 3.5 (2.3), *p* = 0.088). Interestingly, patients treated with etanercept tended to achieve higher OT levels against serotype 3 (57.9% of patients on etanercept vs. 42.3% of subjects on other biologics, *p* = 0.052). In fact, almost 40% of patients with an OT response against serotype 3 were treated with etanercept in comparison to patients based in other biological therapies (Figure 3). Remarkably, Rituximab was other biological DMARD that was associated to a good immunological response against pneumococcus with at least 50% of the patients developing functional antibodies against the majority of serotypes investigated (Figure 3). Twenty-six patients (14.3%) did not achieve OT against any of the serotypes studied. None of the biological agents exhibited association with this absence of response.

Methotrexate, which was the most frequent synthetic DMARD used, did not interfere with the immune response in patients treated with biological agents. In this sense, the number of serotypes with positive response, was similar in patients treated or not with methotrexate (median (IQR): 2.3 (2.0) in patients on methotrexate vs. 2.0 (3.0) in those without methotrexate, *p* = 0.730). Similar results were obtained with the rest of the synthetic DMARDs assessed.

Glucocorticoids did not interfere with the immune response to any serotype, nor with the number of serotypes against which OT were achieved (median (IQR): 1.5 (2.0) in patients treated with glucocorticoids vs. 3 (3.0) in patients without them, *p* = 0.135). The small group of five patients who received a daily dose of prednisone higher than 7.5 mg, showed a lowest number of serotypes with OT than subjects untreated with glucocorticoids (median (IQR): 0 (2.0) vs. 3.0 (3.0), *p* = 0.023).

**Figure 4:**
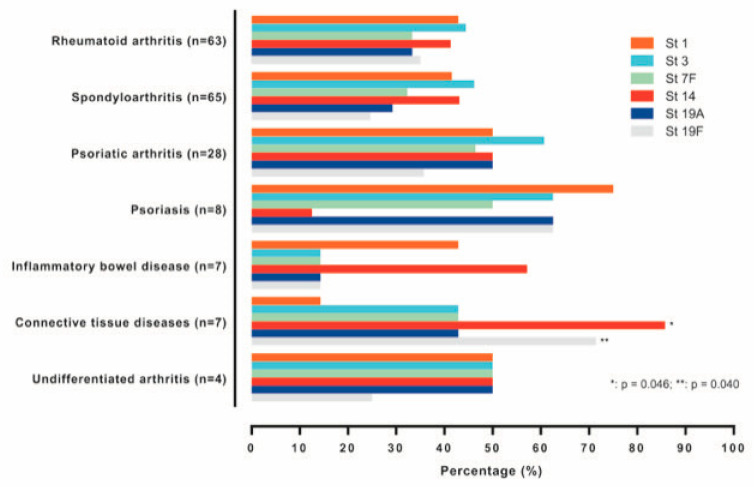
Patients with OT > 50% against each serotype, depending on the diagnosis. Abatacept and anakinra were not included due to the scarce number of patients. St (serotype), OT (opsonization titers).

We also investigated the effect of the diagnosis in the immune response (Figure 4). We found no association between the number of serotypes with OT and diagnosis. The percentages of patients with OT against each of the serotypes studied presented not significant differences, except in the case of subjects with connective tissue diseases, which exhibited a significant higher proportion of responders to serotype 14 (*p* = 0.046) and serotype 19F (*p* = 0.040).

## 4. Discussion

This study evaluates the presence of functional antibodies against the six more prevalent pneumococcal serotypes that are in common between PCV13 and PPV23, which are the two vaccines recommended by different international organizations for immunizing patients with autoimmune diseases treated with biological agents [6,7]. In our study, almost 70% of patients had received PCV13 or PPV23 before being recruited, which is higher than the percentages shown in previous reports [10]. However, only 5% of patients had been vaccinated correctly following the approved guidelines. Considering that all participants came from the hospital out-clinics, this low vaccination coverage probably reflects a deficiency in the design and implementation of vaccination programs for AIID patients that strengthen the communication and collaboration between family physicians and specialists in our community. Our results may help to shed light on the matter of vaccine effectiveness on AIID on biological treatment, thus physicians can make a greater effort to help their patients to increase their adherence to pneumococcal vaccination protocols. 

Vaccination is the best strategy to induce protection against IPD in these kinds of patients treated with DMARDs. Unfortunately, it has been described that patients with autoimmune diseases on immunosuppressive therapy have an impaired immune response to pneumococcal vaccines, being more profound with the conjugate vaccine than with PPV23, because immunosuppressive agents alter largely the cellular immune response generated with the conjugate vaccine [16]. In the current study, the immunological response was measured using an OPKA method to evaluate functional antibodies against the six most prevalent serotypes in adults causing IPD in Spain that are included in both PCV13 and PPV23 vaccines [15]. In 2015, when patients were recruited for this study, 33% of IPD cases were caused by these six serotypes and more importantly, these serotypes were associated to antimicrobial resistance rates accounting for 46% and 32% of reduced susceptibility to penicillin and erythromycin, respectively [15].

Administration of TNF-α blocking agents contributes with a more favorable response compared to patients treated with other immunosuppressive drugs [16,17]. In our study, almost 50% of patients elicited a functional antibody response against at least 50% of the serotypes investigated with a good immune response in patients treated with anti-TNF-α inhibitors such as etanercept for many of the serotypes investigated. Etanercept has been described as not interfering with the humoral response to PPV23 [18]. It has been associated with an immune response to PPV23 in patients with PsoA, similar to that obtained in PsoA patients receiving placebo [19]. Etanercept has also shown a good immune response in RA patients vaccinated with the PCV13 vaccine, not inferior to that obtained in control subjects with osteoarthritis [20]. Therefore, etanercept does not seem to interfere with the antibody production, preserving the humoral response to pneumococcal vaccines. The possible mechanisms explaining this preservation are not well known, but the effect of etanercept on follicular helper T (Tfh) cells, may play a role. Tfh cells represent a small subset of CD4+ helper T cells, which is known to be important during germinal center reaction for production of high-affinity, class-switched antibodies [21]. In this sense, a significant increase in both frequency and absolute numbers of Tfh cells was observed in patients with juvenile idiopathic arthritis treated with etanercept when compared to untreated patients [22].

Treatment with adalimumab in our patients showed a lower response in terms of phagocytic antibodies compared to other biological therapies. These results differ from previous reports where adalimumab showed a good antibody response after vaccination with PPV23 [23]. The difference may be explained in part because Kaine et al. investigated the presence of circulating antibodies and not of functional ones, as we did.

The use of rituximab alone or in combination with methotrexate has also been associated with an impaired immune response in patients with AIIRD [24]. Our results showed that patients treated with rituximab achieved functional phagocytic antibodies against each of the six serotypes analyzed. Hence, possible differences may be due to, first, the antibody determination, as they only measured IgG antibodies, and second, we used the sequential schedule (PCV13 and PPV23 vaccines) to vaccinate our cohort of patients whereas they only used PPV23 vaccine [24]. Another possible bias that could explain the different impact of rituximab on the immune response is the interval period between vaccination and treatment with rituximab. In Bingham’s study, patients received rituximab infusion shortly after vaccination [24]. In our study the majority of pneumococcal vaccines given to patients on rituximab, were administered with the recommended period of 4 weeks before and 6 months after rituximab administration [25], which could explain the higher immune response obtained in our patients treated with rituximab.

Therapy with methotrexate has been associated with reduced vaccine response to pneumococcus because this drug and its metabolites block the novo purine synthesis, affecting the proliferation of B and T cells causing, therefore, a more deleterious effect on the immune response [17,26]. Our results did not show impaired effects of synthetic DMARDs. Patients with and without therapy with methotrexate did not have differences in the OT for each of the six serotypes analyzed. The use of ELISA that quantify IgG levels or OPKA to measure functional killing antibodies, and differences among pneumococcal vaccines administered (PPV23, PCV13 or the sequential PCV13-PPV23) may influence the outcome of the immune response.

Administration of glucocorticoids affects antibody levels in patients undergoing immunosuppressive treatment for inflammatory diseases. The use of daily high doses of prednisone ≥ 20 mg affected negatively the antibody response to PPV23 and in those patients who responded, antibody levels decayed rapidly after vaccination [27]. In our study, although the number of patients with doses of prednisone ≥ 7.5 mg was scarce to achieve any conclusion, they had an impaired immune response of phagocytic antibodies, suggesting that even with a lower dose of prednisone, this therapy is detrimental for the immune response after vaccination.

Patients suffering AIID and treated with biological or synthetic DMARD therapies are at great risk for developing IPD. Serotype 3 is included in both PPV23 and PCV13 vaccines and its incidence in adult population is remarkably high despite the use of these vaccines in the population [15,28]. One possible reason to explain it is a low vaccine coverage in immunocompetent adults. However, the aggressive behavior of this particular serotype can be also attributed to microbiological features. This pathogen is able to produce, express and release a high amount of capsular polysaccharides that saturate anti-serotype 3 specific Ab, reducing opsonophagocytic killing and neutralization, that allows the bacterium to avoid very efficiently the host immune response [29]. Our findings indicate that almost 50% of the patients treated with biological therapies, elicited functional phagocytic antibodies against serotype 3 being even higher when etanercept was the therapeutic option. The pneumococcal serotypes chosen in our study for OPKA analysis, harbored multidrug resistance (MDR) that is a critical point, as these strains might be associated to treatment failure. Vaccination is essential because the host immune response against pneumococcus is boosted by the cooperation of certain antibiotics and specific antibodies [30]. In this sense, β-lactams and macrolides increase complement-mediated immunity and phagocytosis of MDR pneumococcal strains in the presence of functional phagocytic antibodies. Alteration of cell surface structures by these antibiotics even at subinhibitory concentrations results in greater exposure of microbial ligands that are normally hidden or hardly exposed. This alteration of the bacterial envelope stimulated opsonization by functional antibodies and acute phase proteins, increasing the recognition of *S. pneumoniae* by circulating phagocytes [31,32,33]. This is important for vaccinated patients with AIIRD and treated with biological therapies because although the level of antibodies may not be fully protective in many of them, the efficacy of antibiotic treatment in case of developing IPD would be much higher than in unvaccinated patients due to this immunomodulatory effect with antibiotics.

When we looked for some difference in immune response depending on the diagnosis, we found that a group of seven patients with connective tissue diseases showed a significantly better response to serotype 14 and serotype 19F, when compared with subjects with other diagnoses. Despite this, the small sample size, explained mainly because the number of biological agents approved for the treatment of connective tissue disease is low, is too small to draw any conclusion.

We studied the immune response to recommended pneumococcal vaccination in patients with AIID using an opsonophagocytic assay that allows us to evaluate the functional Ab titers, which may be more relevant than knowing the circulating Ab titers. However, as studies using this same method are scarce, it is difficult to establish comparison between them and achieve conclusions that are more powerful. The number of patients with certain treatments is too little to achieve any conclusion, but our sample reflects the reality of the daily out clinics.

## 5. Conclusions

Overall, our study shows that patients with autoimmune inflammatory diseases treated with biological agents, including rituximab, had a functional antibody phagocytic response after a correct program of vaccination using PCV13 and PPV23. These results reinforce the importance of increasing the coverage rates of pneumococcal vaccines in these patients.

## Figures and Tables

**Table 1:** Demographic data, diagnosis, disease duration, biological therapy, and concomitant treatment with synthetic disease modifying antirheumatic drugs (DMARDs) and/or glucocorticoids, of the 182 participants.

Group	n (%)	Age (Mean ± SD)	Gender, n (%) Females	Disease Duration (Mean ± SD), in Years
Whole cohort	182 (100)	50 ± 13	109 (59.9)	9.4 ± 8.8
Diagnosis				
Spondyloarthritis	65 (35.7)	46 ± 11	28 (43.1)	9.8 ± 10.0
Rheumatoid arthritis	63 (34.6)	55 ± 12	50 (79.4)	9.9 ± 7.9
Psoriatic arthritis	28 (15.3)	50 ± 12	14 (50.0)	7.6 ± 8.1
Psoriasis	8 (4.4)	46 ± 10	5 (62.5)	17.0 ± 10.5
Inflammatory bowel disease	7 (3.9)	41 ± 11	3 (42.9)	7.9 ± 6.6
Connective tissue diseases *	7 (3.9)	54 ± 16	7 (100.0)	2.4 ± 2.1
Undifferentiated arthritis	4 (2.2)	33 ± 11	2 (50.0)	4.7 ± 7.2
Biological DMARDs				
Etanercept	57 (31.3)	49 ± 14	30 (52.6)	6.3 ± 5.4
Adalimumab	54 (29.7)	50 ± 10	29 (53.7)	10.8 ± 9.5
Infliximab	21 (11.5)	49 ± 13	13 (61.9)	15.1 ± 12.7
Golimumab	17 (9.3)	49 ± 11	9 (52.9)	8.6 ± 7.9
Certolizumab	6 (3.3)	56 ± 10	3 (50.0)	5.5 ± 3.7
Rituximab	14 (7.7)	56 ± 11	12 (85.7)	10.9 ± 9.6
Tocilizumab	10 (5.5)	49 ± 17	10 (100.0)	7.9 ± 7.3
Abatacept	2 (1.1)	41 ± 16	2 (100.0)	9.8 ± 5.9
Anakinra	1 (0.6)	41	1 (100.0)	5.5
Synthetic DMARDs				
Methotrexate	55 (30.2)	51 ± 14	38 (69.1)	9.3 ± 7.8
Leflunomide	15 (8.2)	56 ± 14	10 (66.7)	8.2 ± 6.8
Sulfasalazine	3 (1.6)	50 ± 11	1 (33.3)	7.5 ± 10.6
Azathioprine	2 (1.1)	34 ± 15	2 (100.0)	7.0 ± 7.0
Hydroxychloroquine	1 (0.6)	50.8	1 (100.0)	4.8
Cyclosporine	1 (0.6)	51.8	1 (100.0)	15.8
Glucocorticoids				
Prednisone ≤7.5 mg/d	23 (12.6)	55 ± 16	18 (78.3)	10.6 ± 9.0
Prednisone >7.5 mg/d	5 (2.7)	49 ± 14	3 (60.0)	3.0 ± 4.2

*: includes 1 systemic sclerosis, 1 Still disease, 1 amyloidosis, 1 ANCA-associated vasculitis, 1 rheumatic polymyalgia, 1 Sjögren syndrome, 1 panuveitis. DMARDs: disease modifying antirheumatic drugs. Variables with just 1 case show absolute values.

**Table 2:** Number and percentage of patients that presented final OT ^1^ against each one of the six serotypes studied.

Pneumococcal Serotypes	Patients with OT n (%)
1	80 (44.0)
3	86 (47.3)
7F	65 (35.7)
14	81 (44.5)
19A	65 (35.7)
19F	60 (33.0)

^1^ OT: opsonization titers.

**Table 3:** Number and percentage of patients that presented final OT ^1^ against each one of the six serotypes studied.

Pneumococcal Serotypes	Patients with OT n (%)
0	26 (14.3)
1	40 (22.0)
2	27 (14.8)
3	39 (21.4)
4	28 (15.4)
5	20 (11.0)

^1^ OT: opsonization titers.

## Data Availability

Data are contained within the article. Additional details are available upon request from the corresponding P.R. (ORCID 0000-0003-2277-6814) and J.Y. (ORCID 0000-0001-7996-0837).
